# Investigations of Alkaline and Enzymatic Membrane Cleaning of Ultrafiltration Membranes Fouled by Thermomechanical Pulping Process Water

**DOI:** 10.3390/membranes8040091

**Published:** 2018-10-10

**Authors:** Gregor Rudolph, Herje Schagerlöf, Kristian B. Morkeberg Krogh, Ann-Sofi Jönsson, Frank Lipnizki

**Affiliations:** 1 Department of Chemical Engineering, Lund University, Naturvetarvägen 14, Box 124, 22100 Lund, Sweden; Herje.Schagerlof@chemeng.lth.se (H.S.); Ann-Sofi.Jonsson@chemeng.lth.se (A.-S.J.); Frank.Lipnizki@chemeng.lth.se (F.L.); 2 Novozymes A/S, Krogshoejvej 36, 2880 Bagsvaerd, Denmark; KBK@novozymes.com

**Keywords:** ultrafiltration, membrane fouling, enzymatic membrane cleaning, alkaline membrane cleaning, pulp and paper industry, biorefinery

## Abstract

The pulp and paper industry is one of the most important industrial sectors worldwide, and has considerable potential for the sustainable fractionation of lignocellulosic biomass to provide valuable compounds. Ultrafiltration (UF) is a suitable separation technique for the profitable production of hemicelluloses from process water from thermomechanical pulping (ThMP), but is limited by membrane fouling. Improvements in cleaning protocols and new alternative cleaning agents are required to ensure a long membrane lifetime, and thus a sustainable process. This study, therefore, focuses on the cleaning of polymeric UF membranes after the filtration of ThMP process water, comparing alkaline with enzymatic cleaning agents. The aim was to develop a cleaning procedure that is efficient under mild conditions, resulting in a lower environmental impact. It was not possible to restore the initial permeability of the membrane when cleaning the membrane with enzymes alone, but the permeability was restored when using a two-step cleaning process with enzymes in the first step and an alkaline cleaning agent in the second step. Scanning electron microscopy gave a deeper inside into the cleaning efficiency. Attenuated total reflectance Fourier-transform infrared spectroscopy analysis confirmed that not only polysaccharides, but also extractives are adsorbed onto the membrane surface.

## 1. Introduction

Pulp and paper mills have considerable potential for the sustainable separation and valorization of components in lignocellulosic biomass in integrated forest biorefineries (IFBRs) [1]. This will be key in a future biobased society, where biomass will be a central resource. The transformation of pulp and paper mills into IFBRs requires energy-efficient separation processes, and processes such as membrane filtration, that do not use heat, could make these separations more energy-efficient [2]. Membrane filtration has been used for the separation of various value-added chemicals in IFBRs [1]. It has been used to concentrate and purify hemicelluloses in thermomechanical pulping (ThMP) process water [3,4], to purify ground wood pulp and paper mill circulation water [5], to treat bleach plant effluent [6], and to reduce the load on the recovery boiler by the recovery of lignin in black liquor [7].

The single most important challenge in membrane filtration is membrane fouling [8]. Membrane fouling reduces the flux and changes the separation characteristics of the membrane. Chemical cleaning is usually used to remove foulants, common cleaning agents being alkaline or acid agents, detergents, oxidants, and enzymes. The choice of cleaning protocol is usually based on experience from similar applications and laboratory experiments. However, a comprehensive literature review of research on the cleaning of ultrafiltration (UF) membranes has shown that far from all cleaning procedures are successful [9].

Membranes in pulp mill biorefineries are mostly exposed to organic foulants that are usually removed by alkaline cleaning. However, alkaline cleaning is carried out under harsh conditions, for example, high pH, which causes membrane aging, which in turn leads to changes in the molecular weight cutoff and flux [9,10,11]. The lifetime of the membrane is also reduced, and frequent membrane replacement is needed, causing plant downtime and increased operating costs. Enzymatic cleaning, on the other hand, can be performed under milder cleaning conditions, and therefore causes less aging of the membrane. It is also possible to tailor enzymatic cleaning agents to the specific conditions in each application, for example, in the removal of foulants during whey ultrafiltration [12,13], the cleaning of UF membranes fouled by abattoir effluent [14], and cleaning of UF membranes after interaction with humic acid [15].

Ultrafiltration has been used to recover hemicelluloses in ThMP process water in several studies [3,4,16,17]. This process water contains mainly hemicelluloses, but also inorganic material, lignin, and small amounts of extractives such as resin or fatty acids. Analysis of membranes fouled by ThMP process water indicates that mainly polysaccharides and extractives are adsorbed on the membrane [18,19]. Cleaning agents containing enzymes that degrade polysaccharides or extractives may thus be suitable for membrane cleaning in thermomechanical pulp mill biorefineries. However, in an initial study, Maartens et al. [20] reported that enzymatic cleaning had no positive impact on the flux recovery of a UF membrane used to filter a Kraft paper mill effluent; in fact, the flux was lower after enzymatic cleaning, as it seems the enzymes themselves adsorbed onto the membranes. Meanwhile, in other industries, e.g., dairy production or wastewater treatment, enzymatic cleaning agents are becoming more established [12,13,21]. 

This study was performed to investigated whether enzymatic cleaning agents could provide an alternative to alkaline cleaning agents for polymeric membranes fouled by ultrafiltration of ThMP process water. Two alkaline cleaning agents (sodium hydroxide and Ultrasil 10) and an enzyme cocktail consisting of polysaccharide- and extractive-degrading enzymes were used. Two-step membrane cleaning was studied, and the cleaning efficiency was determined as the recovery of the membrane permeability after cleaning. Membrane samples were examined using scanning electron microscopy (SEM) and analyzed with attenuated total reflectance Fourier-transform infrared spectroscopy (ATR-FTIR). The water contact angle was also measured to investigate possible changes in hydrophilicity. The most promising cleaning protocols were investigated further in a long-term study involving four filtration and cleaning cycles.

## 2. Materials and Methods 

### 2.1. Process Water

A batch of process water (Kvarnsveden Mill, Stora Ensos, Sweden) was used to foul polymeric UF membranes. The ThMP process water is relatively dilute, with a concentration of less than 1% (*w*/*w*) total solids. Apart from hemicelluloses, it contains residues of lignin, salts, and extractives. Representative concentrations of the main compounds in the ThMP process water are given in Table 1. The process water was stored as frozen and was usually thawed immediately before filtration experiments, and always less than a week before the experiment.

### 2.2. Cleaning Agents and Protocols

Sodium hydroxide (NaOH) (AppliChem GmbH, Darmstadt, Germany), Ultrasil 10 (Ecolab Deutschland GmbH, Monheim am Rhein, Germany), and an enzyme cocktail containing a mixture of enzymes that degrade wood-derived compounds (EC) (Novozymes A/S, Bagsværd, Denmark) were used as cleaning agents.

The enzyme cocktail contains enzymes from six commercially available enzyme classes. Each enzyme was at a concentration of 0.1% (*w*/*v*) in a citrate buffer (pH 5.8). B-glucanase, cellulase, mannanase, and xylanase are polysaccharide-degrading enzymes, lipase is a lipid-degrading enzyme, and cutinase is a hydrolase acting on carboxylic ester bonds. The latter two enzymes are intended to degrade extractives. Concentrations and conditions are a compromise between the suitable conditions for each enzyme.

The following two-step membrane cleaning protocols were applied:

1. Alkaline cleaning with NaOH (NaOH + NaOH).
2. Alkaline cleaning with Ultrasil 10 (U10 + U10).
3. Enzymatic cleaning with the enzyme cocktail followed by NaOH (EC + NaOH).
4. Enzymatic cleaning with the enzyme cocktail followed by Ultrasil 10 (EC + U10).

Two of the cleaning protocols were investigated further in a long-term study with four filtration–cleaning cycles. In these, a membrane was fouled by filtration of ThMP process water for four times. The U10 + U10 protocol was used to clean the membrane after the 1st, 2nd, and 4th cycles of filtration, and the EC + U10 protocol after the 3rd filtration cycle.

### 2.3. Membrane and Equipment

New membranes from the same batch of the commercial polysulfone (PSU) membrane UFX5-pHt (Alfa Laval AB, Nakskov, Denmark) were used in all experiments. This type of membrane is modified so as to be permanently hydrophilic. The nominal molecular weight cutoff is 5 kDa.

All experiments were conducted in a stirred dead-end module with an effective membrane area of about 32 cm^2^. The module was pressurized using nitrogen gas. Stirring and heating was achieved with a magnetic stirrer and heating plate. Permeate was collected in a beaker on an electronic balance, and the weight was recorded continuously. The flux through the membrane was calculated by recording the increase in permeate mass over time, assuming a density of 1000 g/L.

### 2.4. Experimental Procedures

New membrane samples were used in all experiments. Each membrane sample was initially conditioned with 400 mL of 1% (*w*/*v*) Ultrasil 10 solution (for process parameters, see Table 2) at 50 °C to remove preservation chemicals such as glycerine from the pristine membrane. The cleaning solution was then concentrated at 2 bar to achieve a volume reduction (VR) of 50% (i.e., 200 mL of the cleaning solution was withdrawn as permeate). The conditioned membrane was rinsed with a total of 1200 mL deionized water, and the initial membrane permeability was determined from the pure water flux (PWF). Each rinsing cycle consisted of stirring 400 mL deionized water at 50 °C at a rotational speed of 500 rpm and ambient pressure for 1 min. Then, the module was emptied, and rinsing was repeated with another 400 mL of deionized water at approximately 35 °C and a further 400 mL at 20 °C, both at 2 bar and 500 rpm to a VR of 50%. The PWF was measured by filtering deionized water at four pressures: 0.5 bar, 1.0 bar, 1.5 bar, and 2.0 bar; a temperature of 30 °C; and a stirring speed of 500 rpm. The permeate was collected for 5 min at each pressure. 

The membrane permeability (L/m^2^/h/bar) was calculated by dividing the PWF by the applied pressure, and the average was calculated for all four pressures. The membrane permeability after each experiment was normalized to the permeability after conditioning of the new membrane sample.

#### Membrane Fouling and Cleaning

The conditioned membranes were fouled by filtering approximately 3.5 L of ThMP process water at 75 °C, 2 bar, and 500 rpm until about 3 L permeate had been withdrawn. This corresponds to a VR of 88%. The membranes were then rinsed with a total of 1200 mL deionized water as described above to remove reversible fouling.

The fouled and rinsed membranes were cleaned at 50 °C using the cleaning agents listed in Table 2. For the alkaline cleaning, a volume of 400 mL of solution was used during each cleaning cycle. The cleaning solution was then concentrated at 2 bar to a VR of 50%, and the module emptied. 

Afterwards, the membrane was rinsed with a total of 1200 mL as described above, and the PWF of the cleaned membrane was then measured and the permeability calculated.

A volume of 100 mL of the enzyme cocktail was used. After enzymatic cleaning, the membranes were rinsed as previously, but were additionally thoroughly washed with deionized water at 20 °C for 3 min between each rinsing.

### 2.5. Analysis

The membrane samples were dried and stored at 30 °C until examination with SEM, analysis with ATR-FTIR spectroscopy, and water contact angle measurements. 

Images of dried membrane samples were obtained by SEM. Samples measuring 5 × 5 mm^2^ were coated for 3 min with a gold–palladium mixture at a current of 150 mA and a pressure of 7 × 10^−2^ mbar under an argon atmosphere. Then, three spots on each sample were examined in a JSM 6700F NT microscope (JEOL, Sollentuna, Sweden) at an acceleration voltage of 10 kV, and the image containing most of the attributes observed at the three examined spots was chosen.

ATR-FTIR spectra were obtained with an ALPHA FTIR spectrometer (Bruker, Frederikssund, Denmark). The transmittance from a 5 × 5 mm^2^ membrane area was measured in the interval of 400 cm^−1^ to 4500 cm^−1^. At least two measurements were performed on different parts of each membrane sample. Before the membrane sample itself was analyzed, a background signal was obtained from polyethylene terephthalate.

The water contact angle was determined with a Drop and Bubble Shape Tensiometer PAT-1 (SINTERFACE Technologies, Berlin, Germany) and the software PAT-1 (SINTERFACE Technologies, version 8.01.23, Berlin, Germany), where a 6-mL drop of Millipore water was dropped onto a membrane sample (area of approximately 10 × 10 mm^2^).

## 3. Results and Discussion

### 3.1. Two-Step Cleaning Protocols

**Figure 1:**
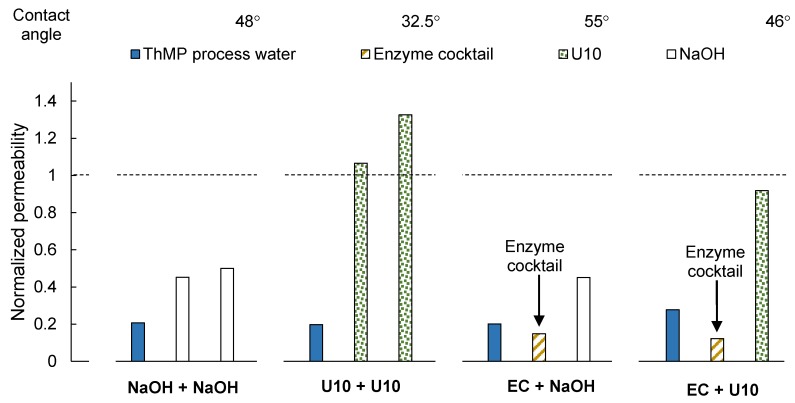
Normalized membrane permeability after fouling with ThMP process water and subsequent two-step cleaning. The water contact angles of the cleaned membrane samples are given above the figure. U10: Ultrasil 10, EC: Enzyme cocktail.

Each membrane sample was fouled by filtration of ThMP process water and subsequently cleaned in a two-step process. The permeability was drastically reduced after the filtration and before cleaning, as presented in Figure 1. The normalized permeability after filtration of ThMP process water was between 0.2 and 0.3.

#### 3.1.1. Alkaline Cleaning

After the first cleaning step with U10, the permeability was higher than that of the pristine, conditioned membrane, and increased further after the second step (U10 + U10 in Figure 1). This is believed to be the result of efficient cleaning in combination with further removal of preservation chemicals, such as glycerin, from the membrane. The permeability was partially recovered when using the cleaning protocol of NaOH + NaOH. However, the normalized permeability after cleaning was only around 0.5, even after the second cleaning step.

The hydrophilicity of the membrane, expressed in terms of the contact angle, increased after alkaline cleaning, and was markedly higher after cleaning with U10: 32.5° versus 48° after cleaning with NaOH. The contact angle of the pristine, conditioned membranes was 71.5°. Since the concentration of NaOH was the same in both alkaline cleaning solutions, the difference in cleaning efficiency is most likely a result of the detergent (SDS) or the chelating agent (EDTA) in Ultrasil 10.

#### 3.1.2. Enzymatic Cleaning

Generally, the permeability after cleaning with the enzyme cocktail was lower than before cleaning (Figure 1). This was also observed when cleaning a pristine, conditioned membrane with the enzyme cocktail (see Figure S1 in the Supplementary Materials). This is because enzymes were adsorbed either on the membrane or on top of the fouling layer on the membrane. Maartens et al. [20] also found that in some cases, it appeared as if the cleaning enzymes themselves were adsorbed onto the membranes, causing additional fouling when cleaning UF membranes after fouling with a pulp and paper mill effluent. 

Applying NaOH or U10 in the second step improved the permeability. The normalized permeability was 0.45 after cleaning with NaOH and 0.92 after cleaning with U10. This is in accordance with previous findings, where the flux increased when detergents were used as a second step after enzymatic cleaning [12].

The normalized permeability after cleaning with EC + NaOH was similar to that after the first cleaning step in the NaOH + NaOH protocol, and the permeability after cleaning with EC + U10 was similar to that after the first cleaning step in the U10 + U10 protocol. It thus appears as if the first cleaning step with the enzyme cocktail had no foulant removal effect and the recovery of the permeability after the second cleaning step was due only to the alkaline cleaning agents. Therefore, further analysis was performed.

#### 3.1.3. SEM Analysis

**Figure 2:**
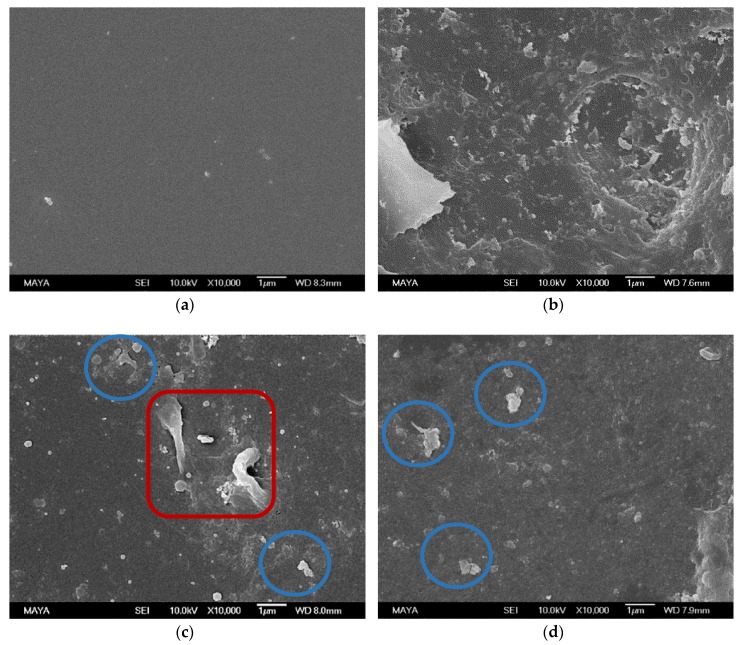

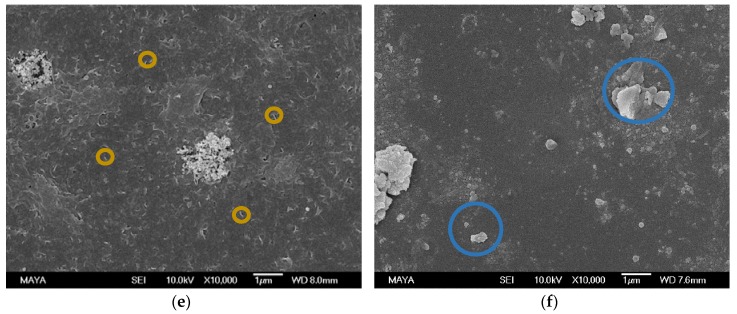
SEM images of membrane samples after: (**a**) conditioning, (**b**) fouling with ThMP process water, (**c**) NaOH + NaOH cleaning, (**d**) U10 + U10 cleaning, (**e**) EC + NaOH cleaning, and (**f**) EC + U10 cleaning. Objects assignment: assumingly extractives (circle in blue), assumingly enzymes (circle in brown), unassigned (red square).

A remarkable difference was found between the surface of a pristine, conditioned membrane (Figure 2a) and a fouled membrane (Figure 2b). The pristine state was partly recovered when using the NaOH + NaOH (Figure 2c), U10 + U10 (Figure 2d), and EC + U10 (Figure 2f) cleaning protocols, as parts of the smooth membrane surface are visible in these images. Agglomerates (circled in blue) in Figure 2c,d,f can be interpreted as extractives. The large structure visible in Figure 2c (red square), cannot be assigned. The many small objects visible in Figure 2e (circled in brown) seem to be on the same order of magnitude as enzymes found in SEM images of other investigations [24,25]. We therefore interpret them as adsorbed enzymes originating from the enzymatic cleaning step. If the small objects are indeed enzymes, apparently, cleaning with U10 desorbed them from the surface (compared to Figure 2f).

#### 3.1.4. ATR-FTIR Analysis

**Figure 3:**
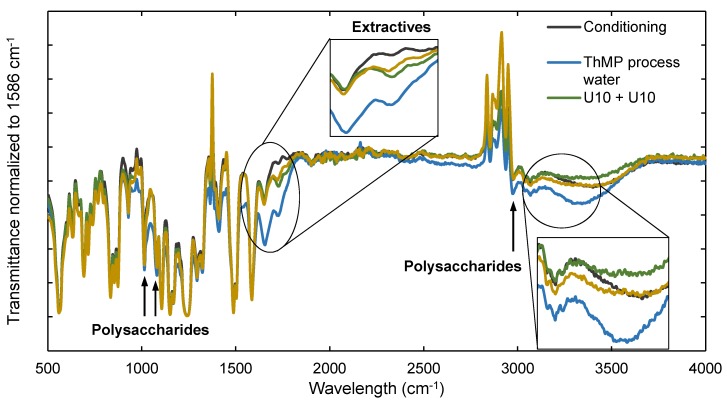
ATR-FTIR spectra of membrane samples after conditioning (gray), filtration of ThMP process water (blue line), two-step alkaline cleaning with Ultrasil 10 (U10 + U10, green), and two-step enzymatic cleaning with the enzyme cocktail followed by Ultrasil 10 (EC + U10, brown). The transmittance was normalized to the intensity of the transmittance peak at 1586 cm^−1^.

Figure 3 shows ATR-FTIR spectra of a conditioned membrane sample, a membrane fouled with ThMP process water, a membrane after cleaning with U10 + U10, and a membrane after cleaning with EC + U10. The band assignments are summarized in Table 3. Each spectrum was normalized to the intensity of the absorbance peak at 1586 cm^−1^, a characteristic peak of PSU. The intensity of this peak should be independent of the treatment of the membrane, and therefore remain constant. Other typical peaks of PSU are found in the bands at 635 cm^−1^ to 855 cm^−1^, 873 cm^−1^, 1157 cm^−1^, 1169 cm^−1^, 1240 cm^−1^, 1295 cm^−1^, 1323 cm^−1^, and 1411 cm^−1^ to 1586 cm^−1^ [26,27,28].

Differences were seen between the conditioned, the fouled, and the cleaned membranes for the peaks at 1014 cm^−1^, 1080 cm^−1^, and 2967 cm^−1^. These wavelengths are assigned as signals of both PSU and polysaccharides [26,28]. These three peaks were attenuated in the two cleaned membranes compared to the fouled membrane, meaning polysaccharides were removed during cleaning. The signal resulting from PSU should not change, thus the difference between the transmittance of the samples can be interpreted as being mainly due to the polysaccharide content on the membrane surface.

However, the difference in transmittance between the samples cleaned with U10 + U10 and EC + U10 is too small to draw reliable conclusions regarding which cleaning procedure was more efficient with regard to polysaccharide removal. The band from 3000 cm^−1^ to 3400 cm^−1^ can result from stretching of O–H groups mainly found in polysaccharides [28,29,30], but also from glycerine, the chemical used for membrane preservation. The spectra from the conditioned membrane and the EC + U10-cleaned membrane are very similar, indicating a removal of polysaccharides by this cleaning protocol. However, the transmittance of the membrane cleaned with U10 + U10 is lower than that of the conditioned membrane, which might be due to the removal of glycerine. If this is the case, then it is uncertain whether the transmittance of the EC + U10-cleaned membrane is higher due to the removal of polysaccharides or also glycerine.

The peak at 1728 cm^−1^ is associated with the C=O stretching of lipophilic extractives, fatty acids, resin acids, steryl esters, and triglycerides. These compounds have been found previously on membranes used for the filtration of pulp and paper mill process water [19]. It shows that extractives also foul the membrane, in addition to polysaccharides. The transmittance of the cleaned membrane samples at this wavelength is not the same as the transmittance of the conditioned membrane sample. However, it is attenuated for the cleaned membrane in comparison with the transmittance of the fouled membrane sample at 1728 cm^−1^. It can therefore be concluded that extractives were partly, but not completely, removed from the membrane.

### 3.2. Long-Term Study

**Figure 4:**
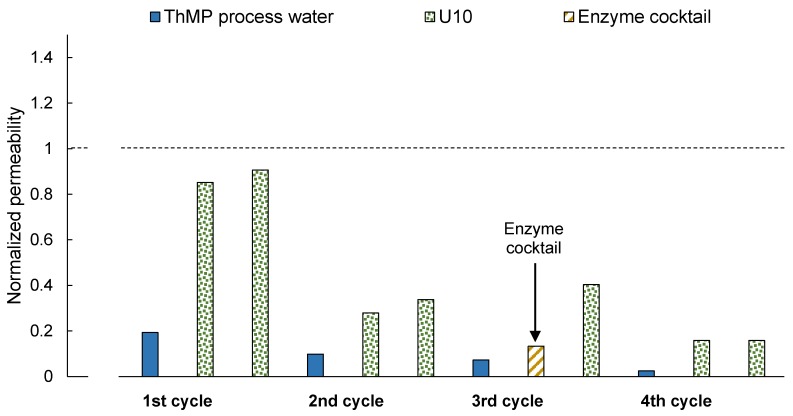
Normalized membrane permeability after fouling with ThMP process water (black) and subsequent two-step cleaning. The unshaded bars show the normalized permeability after cleaning with Ultrasil 10, and the shaded bar after cleaning with the enzyme cocktail. The same membrane sample was used for the complete experiment.

In the long-term study, the membrane permeability decreased successively after each filtration of ThMP process water, as can be seen in Figure 4. Cleaning partly restored the membrane permeability, but a complete recovery of the initial permeability was not achieved.

The water contact angle at the end of the long-term study was rather high, at 65°. This is much higher than for the pristine membrane (71.5°) and after the U10 + U10 cleaning procedure (32.5°, see Figure 1) as well as after the EC + U10 cleaning procedure (46°, see Figure 1). Since extractives are typically hydrophobic, the higher contact angle can most likely originate from an accumulation of extractives on the membrane surface, as was already indicated by the results of the ATR-FTIR analysis (see Figure 3). There thus appears to be some kind of irreversible fouling that reduces the permeability over time.

#### 3.2.1. First and Second Cycles—Two-Step Cleaning U10 + U10

Two-step cleaning with U10 after the first filtration cycle led to the recovery of permeability similar to that after U10 + U10 cleaning, as shown in Figure 1, although the normalized permeability was only 0.85 after the first cleaning step and 0.91 after the second cleaning step. The difference compared to the previous experiment (U10 + U10 in Figure 1) could be caused by a variation in membrane quality or alteration in the ThMP process water. The normalized permeability after the second cycle was only 0.34, which already indicates foulant accumulation.

#### 3.2.2. Third Cycle—Two-Step Cleaning EC + U10

In contrast to previous results (see EC + U10 in Figure 1), the normalized permeability increased already after the first cleaning step with the enzyme cocktail. The second cleaning step with U10 resulted in a normalized permeability of 0.40, which is higher than the permeability the membrane had before the third cycle. This cleaning procedure thus restored the permeability obtained with the previous cleaning step. It can be assumed that mainly polysaccharides were removed during EC + U10 cleaning, as the enzyme cocktail contains mainly polysaccharide-degrading enzymes. This supports the hypothesis suggested by Thuvander et al. [18] and Susanto and Ulbricht [31] that membrane fouling in this application is mainly caused by the adsorption of polysaccharides.

#### 3.2.3. Fourth Cycle—Two-Step Cleaning U10 + U10

The permeability after the fourth and final filtration and cleaning cycle was low, at 0.16, and indicates that U10 does not remove all the foulants of the membrane (compared to Figure 4, second filtration cycle). However, the lower permeability can also be assigned to aging of the ThMP process water. The experimental setup only allowed the addition of fresh ThMP process water to the remaining solution in the feed tank, instead of the emptying of the tank and subsequently replacement of old with fresh ThMP process water after each filtration cycle. This resulted in an accumulation of old ThMP process water in the tank, the properties of which change over time. Aging of ThMP process water was observed in previous experiments when stored for more than several days at room temperature. In the performed study, an experiment took 1.5 weeks.

#### 3.2.4. SEM and Water Contact Angle

**Figure 5:**
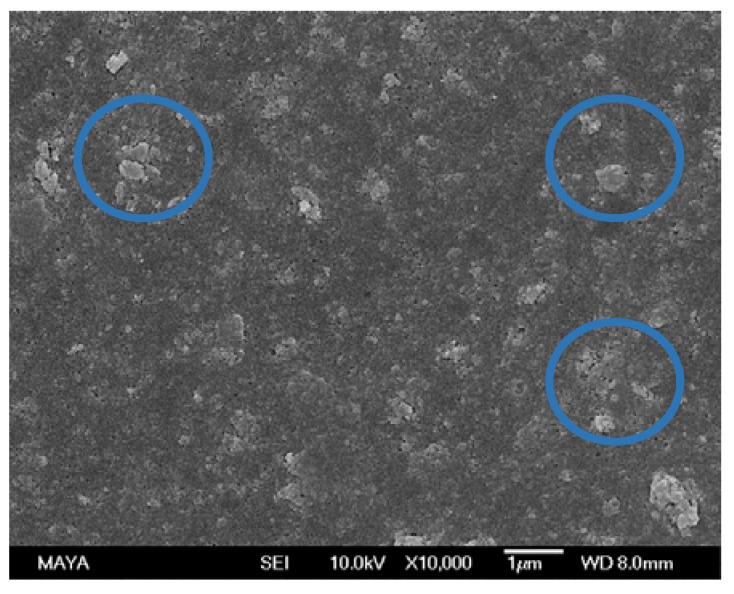
SEM image of a membrane sample after final cleaning in the long-term study. Objects assignment: assumingly extractives (circle in blue).

A SEM image of the membrane sample used in the long-term study (Figure 5) gives similar indications as the ATR-FTIR results and the water contact angle measurements. The image contains the same agglomerates (circled in blue) on the membrane surface as found in Figure 2c,d,f. Hence, these could be interpreted as foulants that are neither removed by enzymatic cleaning nor U10 and are therefore most likely extractives.

The experimental evidence thus points to the accumulation of polysaccharides and extractives on the membrane over time when only cleaned with U10. Enzymatic cleaning with the enzyme cocktail appeared to remove mainly polysaccharides, as shown by an increase in permeability after enzymatic cleaning, but not extractives, as indicated by the increase in water contact angle after the long-term study and the agglomerates found in the SEM images.

## 4. Conclusions

When the membrane was cleaned with an enzyme cocktail containing polysaccharide- and extractive-degrading enzymes, the resulting permeability was lower than that of a fouled membrane after only rinsing it with water. However, subsequent cleaning with Ultrasil 10 restored the membrane permeability almost as efficiently as two-step cleaning with Ultrasil 10. SEM images indicated that the cleaning enzymes became additional foulants on the membrane surface. Some ATR-FTIR bands indicated better removal of polysaccharides after enzymatic cleaning than after two-step Ultrasil 10 cleaning. Both the two-step enzymatic cleaning and two-step Ultrasil 10 cleaning only partly removed extractives from the membrane surface.

In the long-term study, two-step enzymatic cleaning restored the membrane permeability to the permeability observed prior to fouling with process water. It thus appears that polysaccharides are indeed removed by the polysaccharide-degrading enzymes in the cocktail. An increase in water contact angle at the end of the long-term study indicates a reduction in hydrophilicity due to an accumulation of extractives on the membrane surface, even after repeated cleaning with Ultrasil 10. 

Further development of enzymatic cleaning protocols is needed. We suggest that two enzymatic cleaning agents be developed: one attacking polysaccharides, and one attacking extractive foulants. The issue with enzyme adsorption on the membrane surface has to be overcome. Future work will include optimization of the enzyme cocktails as well as cleaning parameters such as temperature and the duration of cleaning. Nevertheless, our findings provide new insight to support the future utilization of enzymatic cleaning agents in thermomechanical pulp mill biorefineries.

## Figures and Tables

**Table 1:** Characteristic composition of the ThMP ^1^ process water. The process water was analyzed in accordance with the standardized NREL ^2^ laboratory analytic procedure [22,23].

Total solids (g/L)	5.4
Ash (g/L)	1.85
Hemicelluloses (g/L)	2.60
Arabinan (g/L)	0.12
Galactan (g/L)	0.35
Glucan (g/L)	0.64
Mannan (g/L)	1.49
Total lignin (g/L)	0.96
Turbidity (NTU ^3^)	307

^1^ Thermomechanical pulping; ^2^ National Renewable Energy Laboratory; ^3^ Nephelometric Turbidity Unit.

**Table 2:** Process parameters for each cleaning agent.

Cleaning Agent	Duration	Stirring	Pressure	Concentration
NaOH	1 h, then VR ^1^ 50%	500 rpm	Ambient, then 2 bar	0.2% (*w*/*v*)
Ultrasil 10 (20% NaOH)	1 h, then VR 50%	500 rpm	Ambient, then 2 bar	1.0% (*w*/*v*)
Enzyme cocktail:				
B-glucanase, cellulose, cutinase, lipase, mannanase, xylanase	3 h	500 rpm	Ambient	0.1% (*v*/*v*) each

^1^ Volume reduction.

**Table 3:** Assignment of attenuate total reflectance-FTIR bands.

Wavenumber (cm^−1^)	Assignment	References
635–875	C–H bending, rocking of PSU ^1^	[28]
1014, 1080	Skeletal aliphatic C–C bonds/aromatic C–H bonds of PSU and polysaccharides	[28,26]
1107, 1169	C–C bonds /aromatic hydrogen bending/rocking of PSU	[26]
1157	C–SO_2_–C symmetric stretching of PSU	[26,27,28]
1240	C–O–C symmetric stretching of PSU	[26,27,28]
1295	S=O stretching of PSU	[26,27,28]
1323	C–SO_2_–C asymmetric stretching of PSU	[28]
1503, 1580, 1586	C=C stretching of PSU in the aromatic rings	[26,28]
1690–1750	C=O stretching of extractives	[19,28]
2967	Aromatic C–H stretching of PSU and polysaccharides	[27]
3000–3400	O–H stretching of polysaccharides	[28]

^1^ polysulphone.

## Supplementary Materials

The following are available online at http://www.mdpi.com/2077-0375/8/4/91/s1, Figure S1: Normalized membrane permeability after fouling with TMP process water as well as after treatment with each cleaning agent. The water contact angles of the cleaned membrane samples are shown above the figure. The contact angle of the conditioned membrane was 71.5°. New membrane samples were used in all experiments.

## References

[1] Bokhary A., Cui L., Lin H.L., Liao B.Q. (2017). A Review of Membrane Technologies for Integrated Forest Biorefinery. J. Membr. Sci. Res..

[2] Sholl D.S., Lively R.P. (2016). Seven chemical separations to change the world. Nature.

[3] Persson T., Jönsson A.-S. (2010). Isolation of hemicelluloses by ultrafiltration of thermomechanical pulp mill process water—Influence of operating conditions. Chem. Eng. Res. Des..

[4] Thuvander J., Jönsson A.-S. (2016). Extraction of galactoglucomannan from thermomechanical pulp mill process water by microfiltration and ultrafiltration—Influence of microfiltration membrane pore size on ultrafiltration performance. Chem. Eng. Res. Des..

[5] Kallioinen M., Huuhilo T., Reinikainen S.-P., Nuortila-Jokinen J., Mänttäri M. (2006). Examination of membrane performance with multivariate methods: A case study within a pulp and paper mill filtration application. Chemometr. Intell. Lab. Syst..

[6] Nordin A.-K., Jönsson A.-S. (2006). Case study of an ultrafiltration plant treating bleach plant effluent from a pulp and paper mill. Desalination.

[7] Keyoumu A., Sjödahl R., Henriksson G., Ek M., Gellerstedt G., Lindström M.E. (2004). Continuous nano- and ultra-filtration of kraft pulping black liquor with ceramic filters. Ind. Crop. Prod..

[8] Cheryan M. (1998). Ultrafiltration and Microfiltration Handbook.

[9] Regula C., Carretier E., Wyart Y., Gésan-Guiziou G., Vincent A., Boudot D., Moulin P. (2014). Chemical cleaning/disinfection and ageing of organic UF membranes: A review. Water Res..

[10] Antón E., Álvarez J.R., Palacio L., Prádanos P., Hernández A., Pihlajamäki A., Luque S. (2015). Ageing of polyethersulfone ultrafiltration membranes under long-term exposures to alkaline and acidic cleaning solutions. Chem. Eng. Sci..

[11] Teella A., Zydney A.L., Zhou H., Olsen C., Robinson C. (2015). Effects of chemical sanitization using NaOH on the properties of polysulfone and polyethersulfone ultrafiltration membranes. Biotechnol. Prog..

[12] Muñoz-Aguado M.J., Wiley D.E., Fane A.G. (1996). Enzymatic and detergent cleaning of a polysulfone ultrafiltration membrane fouled with BSA and whey. J. Membr. Sci..

[13] Argüello M. (2003). Enzymatic cleaning of inorganic ultrafiltration membranes used for whey protein fractionation. J. Membr. Sci..

[14] Maartens A., Swart P., Jacobs E.P. (1996). An enzymatic approach to the cleaning of ultrafiltration membranes fouled in abattoir effluent. J. Membr. Sci..

[15] Yu C.-H., Fang L.-C., Lateef S.K., Wu C.-H., Lin C.-F. (2010). Enzymatic treatment for controlling irreversible membrane fouling in cross-flow humic acid-fed ultrafiltration. J. Hazard. Mater..

[16] Willför S., Rehn P., Sundberg A., Sundberg K., Holmbom B. (2003). Recovery of water-soluble acetylgalactoglucomannans from mechanical pulp of spruce. TAPPI J..

[17] Persson T., Krawczyk H., Nordin A.-K., Jönsson A.-S. (2010). Fractionation of process water inthermomechanical pulp mills. Bioresour. Technol..

[18] Thuvander J., Zarebska A., Helix-Nielsen C., Jönsson A.-S. (2018). Characterisation of irreversible fouling after ultrafiltration of thermomechanical pulp mill process water. J. Wood Chem. Technol..

[19] Puro L., Kallioinen M., Mänttäri M., Nyström M. (2011). Evaluation of behavior and fouling potential of wood extractives in ultrafiltration of pulp and paper mill process water. J. Membr. Sci..

[20] Maartens A., Jacobs E.P., Swart P. (2002). UF of pulp and paper effluent: membrane fouling-prevention and cleaning. J. Membr. Sci..

[21] D’Souza N.M., Mawson A.J. (2005). Membrane cleaning in the dairy industry: A review. Crit. Rev. Food Sci. Nutr..

[22] Sluiter A., Hames B., Ruiz R., Scarlata C., Sluiter J., Templeton D. (2006). Determination of Sugars, Byproducts, and Degradation Products in Liquid Fraction Process Samples; Revised January 2008. https://www.nrel.gov/docs/gen/fy08/42623.pdf.

[23] Sluiter A., Hames B., Ruiz R., Scarlata C., Sluiter J., Templeton D., Crocker D. (2008). Determination of Structural Carbohydrates and Lignin in Biomass; Revised July 2011. https://www.nrel.gov/docs/gen/fy13/42618.pdf.

[24] Li Q., Bi Q.-Y., Lin H.-H., Bian L.-X., Wang X.-L. (2013). A novel ultrafiltration (UF) membrane with controllable selectivity for protein separation. J. Membr. Sci..

[25] Kim J.T., Shin G.H. (2015). Adsorption behavior of β-lactoglobulin onto polyethersulfone membrane surface. J. Adhes. Sci. Technol..

[26] McCutcheon J.R., Hoek E.M.V., Bui N., Lind M.L. (2010). Nanostructured Membranes for Engineered Osmosis Applications. International Patent Application.

[27] Kumar R., Isloor A.M., Ismail A.F., Rashid S.A., Matsuura T. (2013). Polysulfone–Chitosan blend ultrafiltration membranes: preparation, characterization, permeation and antifouling properties. RSC Adv..

[28] Wei X., Wang Z., Wang J., Wang S. (2012). A novel method of surface modification to polysulfone ultrafiltration membrane by preadsorption of citric acid or sodium bisulfite. Membr. Water Treat..

[29] Max J.-J., Chapados C. (2007). Glucose and fructose hydrates in aqueous solution by IR spectroscopy. J. Phys. Chem. A.

[30] Silva F.F., Brites Alves A.M., de Lurdes Serrano M., de Sousa A.P.M. (2017). Isolation and purification of concentrated and non-concentrated hemicellulose alkaline extracts. Sep. Purif. Technol..

[31] Susanto H., Ulbricht M. (2006). Insights into polysaccharide fouling of ultrafiltration membranes. Desalination.

