# Supplementary material for: Investigations of Alkaline and Enzymatic Membrane Cleaning of Ultrafiltration Membranes Fouled by Thermomechanical Pulping Process Water
Source: Membranes (Basel). 2018 Oct 10;8(4):91. doi: 10.3390/membranes8040091 (PMC6316253; doi:10.3390/membranes8040091)
Supplement: Supplementary file 1 [file membranes-08-00091-s001.pdf]

membranes

Supplementary Materials:

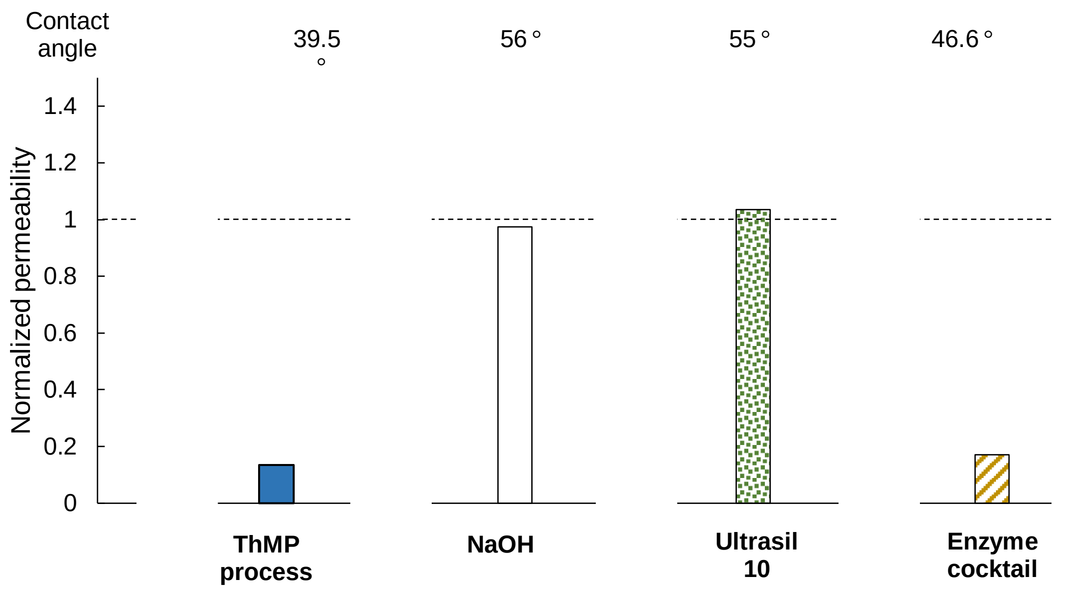

Figure S1. Normalized membrane permeability after fouling with TMP process water as well as after treatment with each cleaning agent. The water contact angles of the cleaned membrane samples are shown above the figure. The contact angle of the conditioned membrane was 71.5°. New membrane samples were used in all experiments.
